# Supplementary figures and images for: Structural and functional characterization of AfsR, an SARP family transcriptional activator of antibiotic biosynthesis in Streptomyces
Source: PLoS Biol. 2024 Mar 1;22(3):e3002528. doi: 10.1371/journal.pbio.3002528 (PMC10936776; doi:10.1371/journal.pbio.3002528)

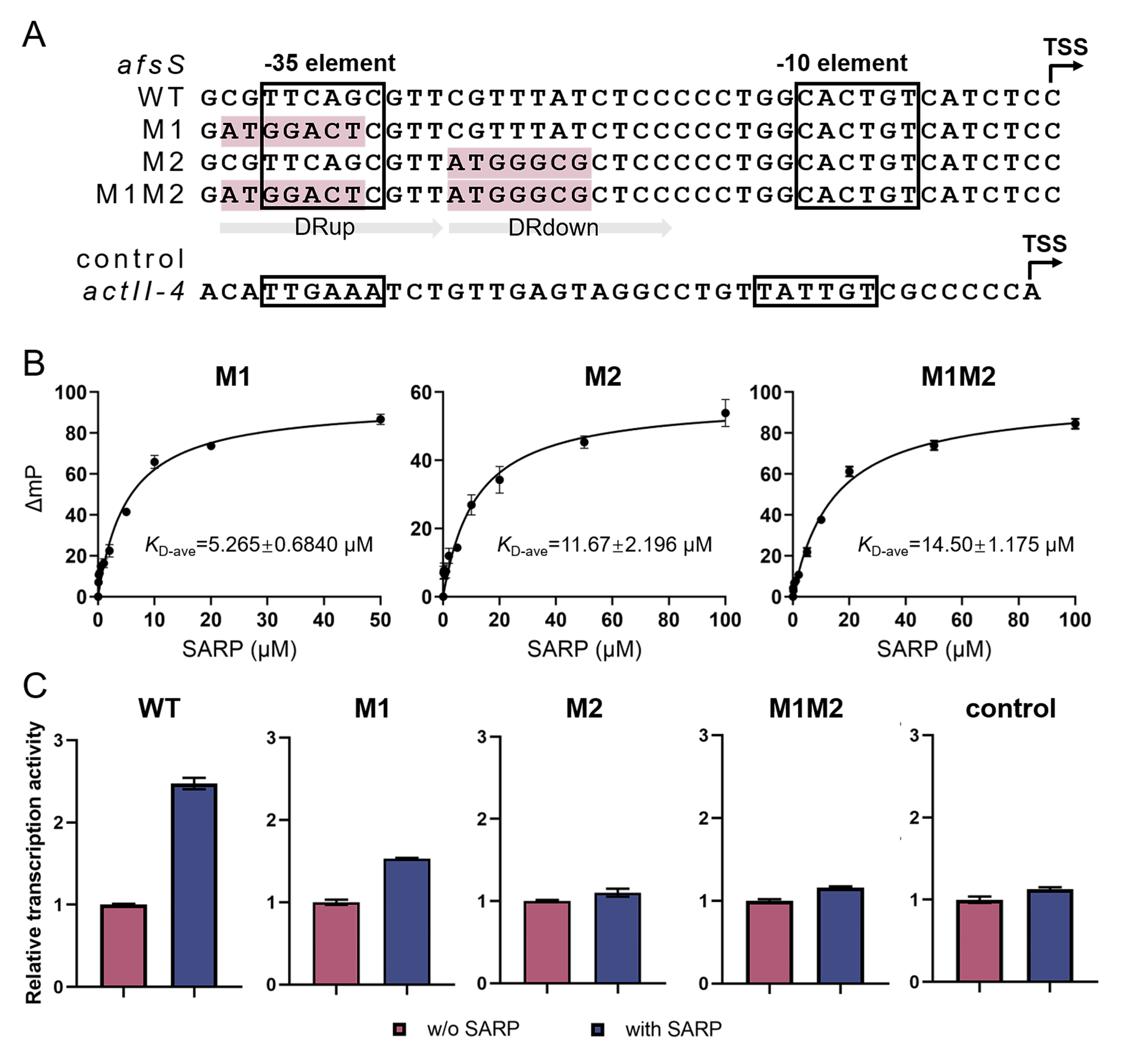

Supplement: S1 Fig — (A) Core promoter sequences used for in vitro assays. The mutations introduced into the afs box are highlighted. The mutated target sites contained mutations in upstream repeat (M1), the downstream repeat (M2), or both repeats (M1M2). The actII-4 promoter was used as a control. (B) Fluorescence polarization assays of the SARP with mutant afs box (M1, M2, and M1M2). Error bars represent mean ± SEM of n = 3 experiments. (C) In vitro MangoIII-based transcription assays with or without 500 nM SARP in the absence of RbpA and CarD. Error bars represent mean ± SEM of n = 3 experiments. The data underlying B and C can be found in S1 Data. (TIF) [file pbio.3002528.s001.tif]

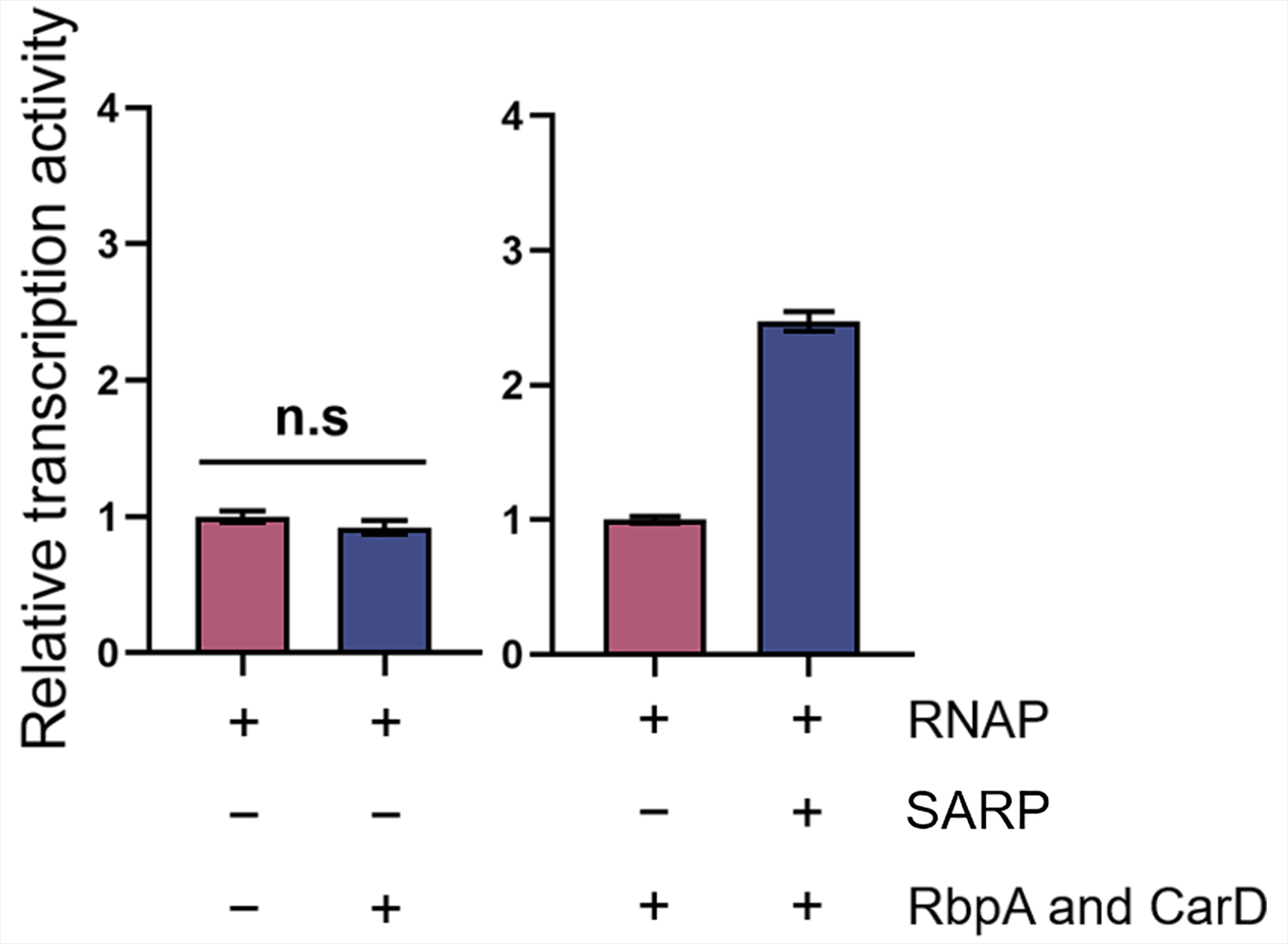

Supplement: S2 Fig — In vitro transcription assays with or without RbpA and CarD on the afsS promoter (left) and transcription assays with or without 500 nM SARP in the presence of RbpA and CarD (right). The data underlying this figure can be found in S1 Data; error bars, SEM; n = 3. (TIF) [file pbio.3002528.s002.tif]

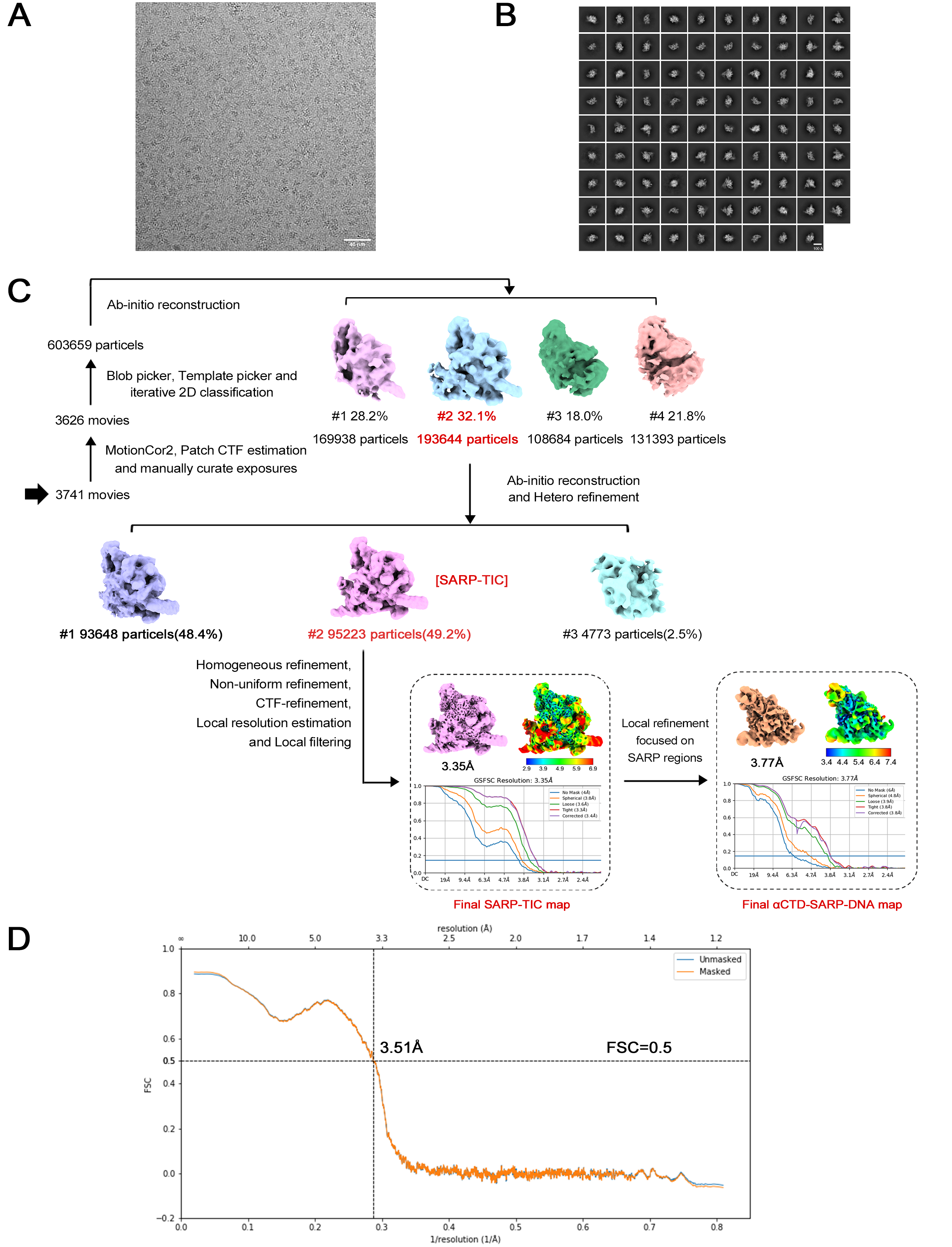

Supplement: S3 Fig — (A) A motion-corrected image (scale bar: 40 nm). (B) 2D classes (scale bar: 100 Å). (C) Data processing pipeline for the dataset of SARP-TIC. The final cryo-EM map of the SARP-TIC was reconstructed using a total of 95,223 single particles and refined to a nominal resolution of 3.35 Å. Local refinement focused on the SARP region generated a 3.77-Å-resolution map. (D) Validation of cryo-EM structural models. Map vs. model FSCs was generated by Phenix (Version 1.19.2). The model-map resolution for the atomic model and cryo-EM map at FSC = 0.5 cutoff was indicated in the figure and reported in S1 Table. The data underlying this figure can be found in S1 Data. (TIF) [file pbio.3002528.s003.tif]

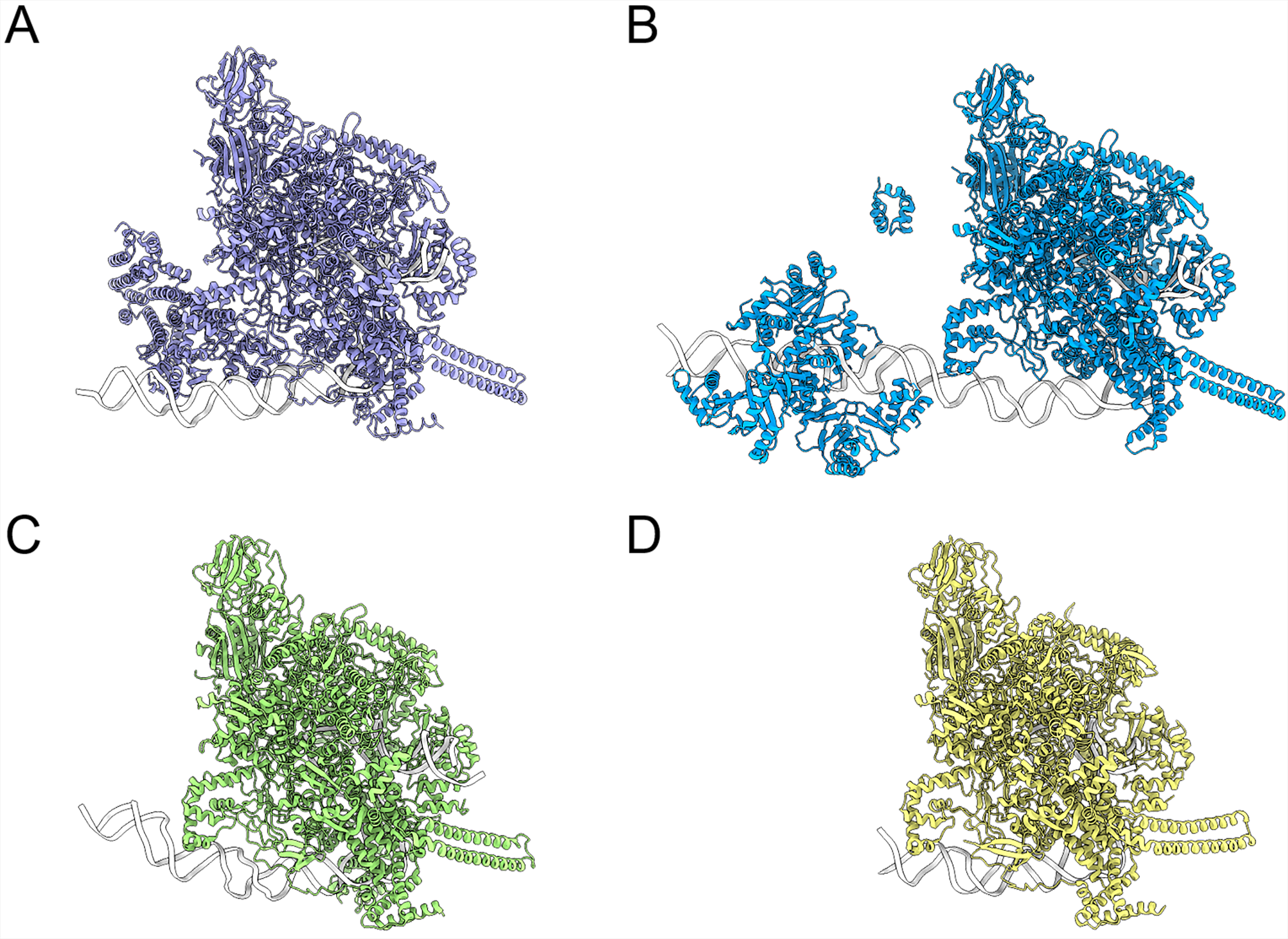

Supplement: S4 Fig — (A) S. coelicolor SARP-TIC. (B) S. coelicolor RNAPσHrdB-Zur-DNA (PDB ID: 7X75). (C) Mycobacterium tuberculosis RNAP-promoter open complex (PDB ID: 6VVY). (D) Mycobacterium smegmatis TIC (PDB ID: 5VI5). (TIF) [file pbio.3002528.s004.tif]

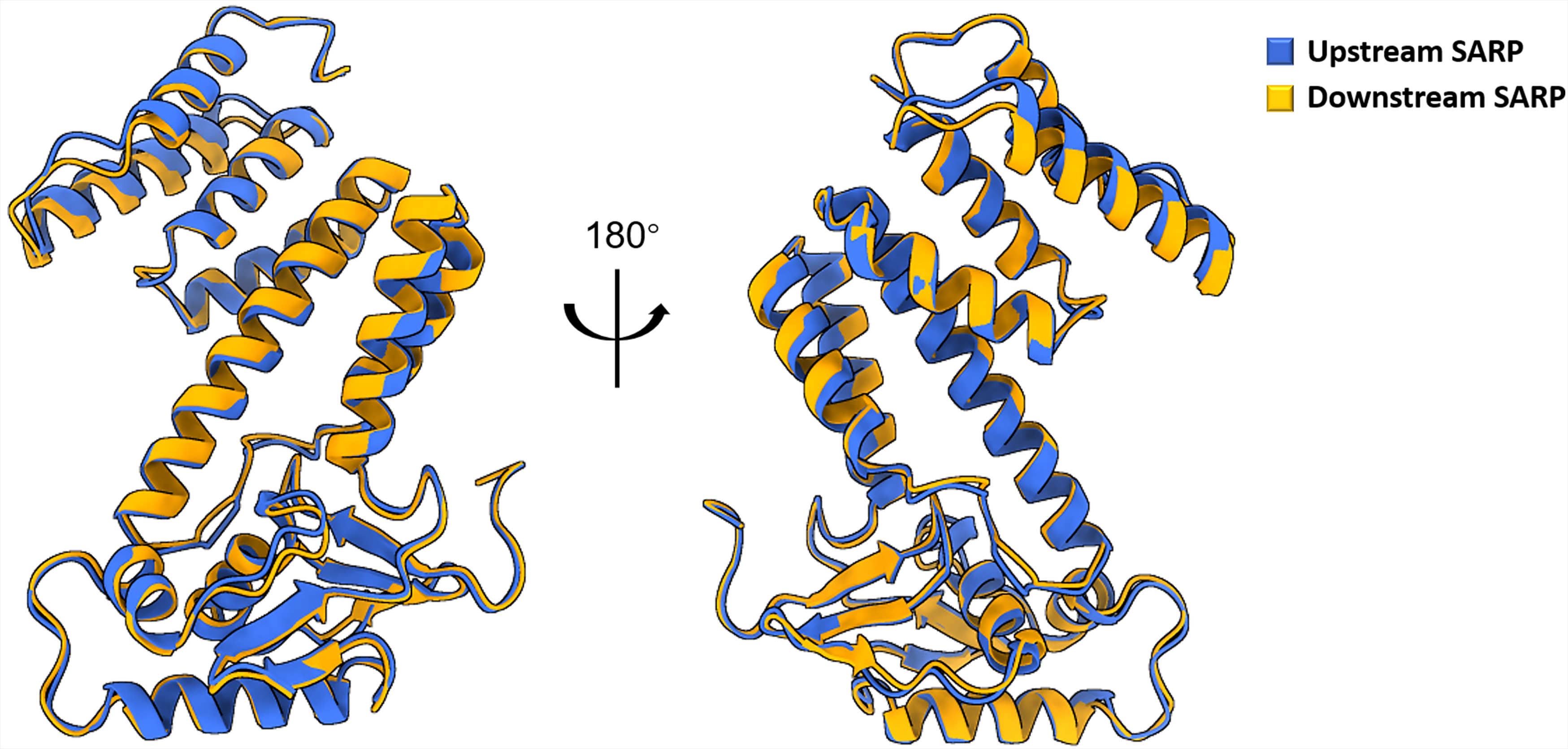

Supplement: S5 Fig — Overall conformations of the 2 protomers are essentially the same with an overall rmsd of 0.8 Å. (TIF) [file pbio.3002528.s005.tif]

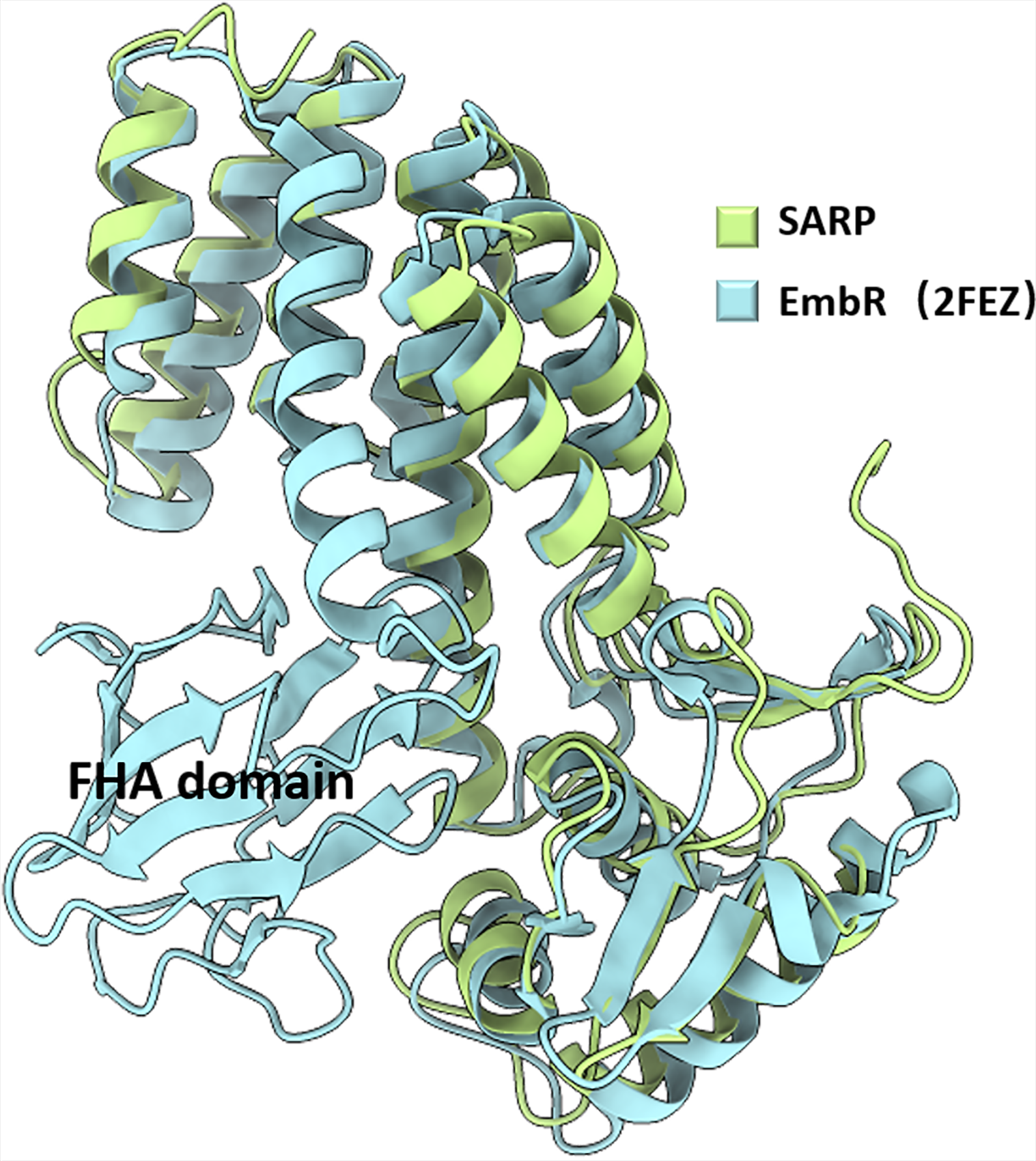

Supplement: S6 Fig — (TIF) [file pbio.3002528.s006.tif]

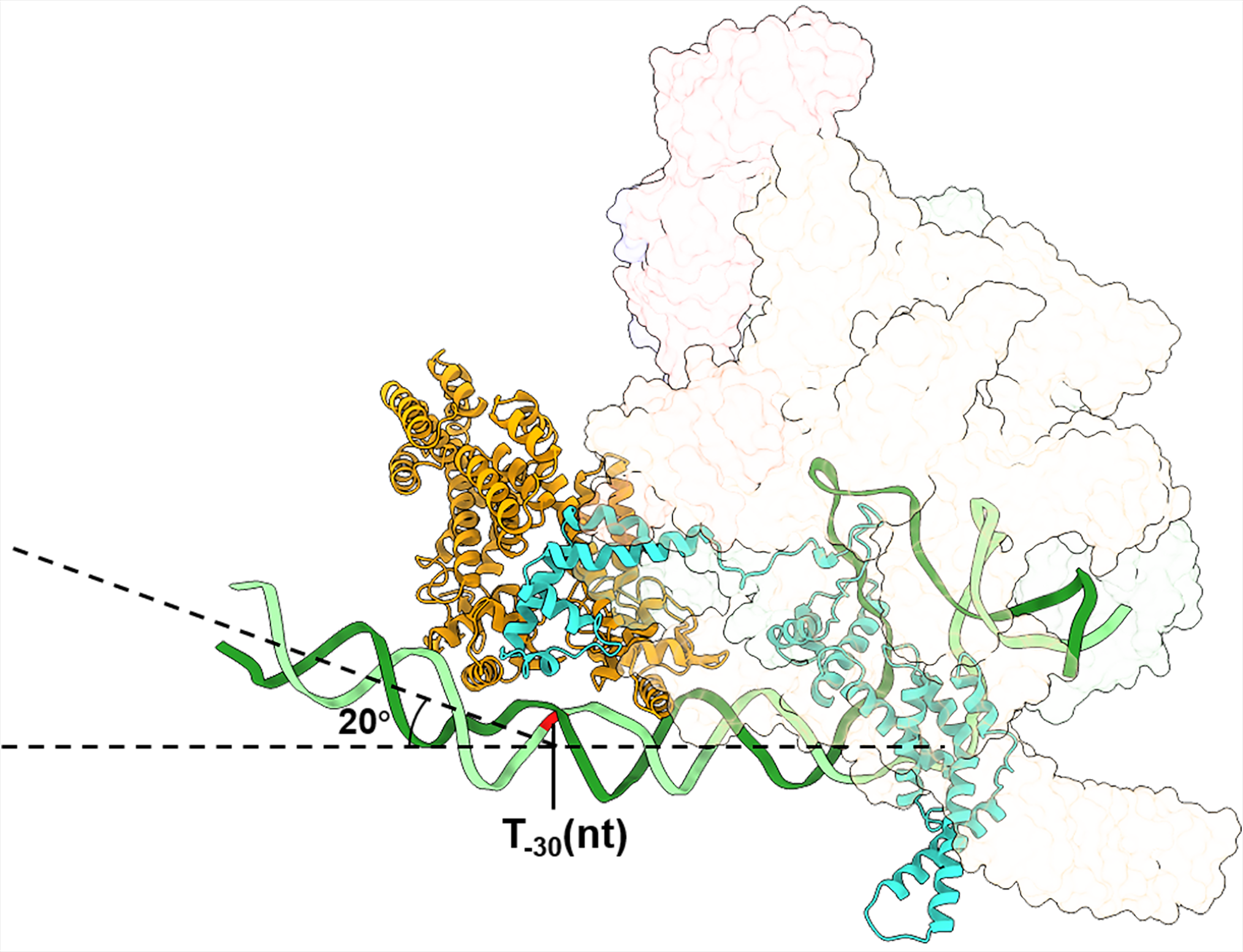

Supplement: S7 Fig — (TIF) [file pbio.3002528.s007.tif]

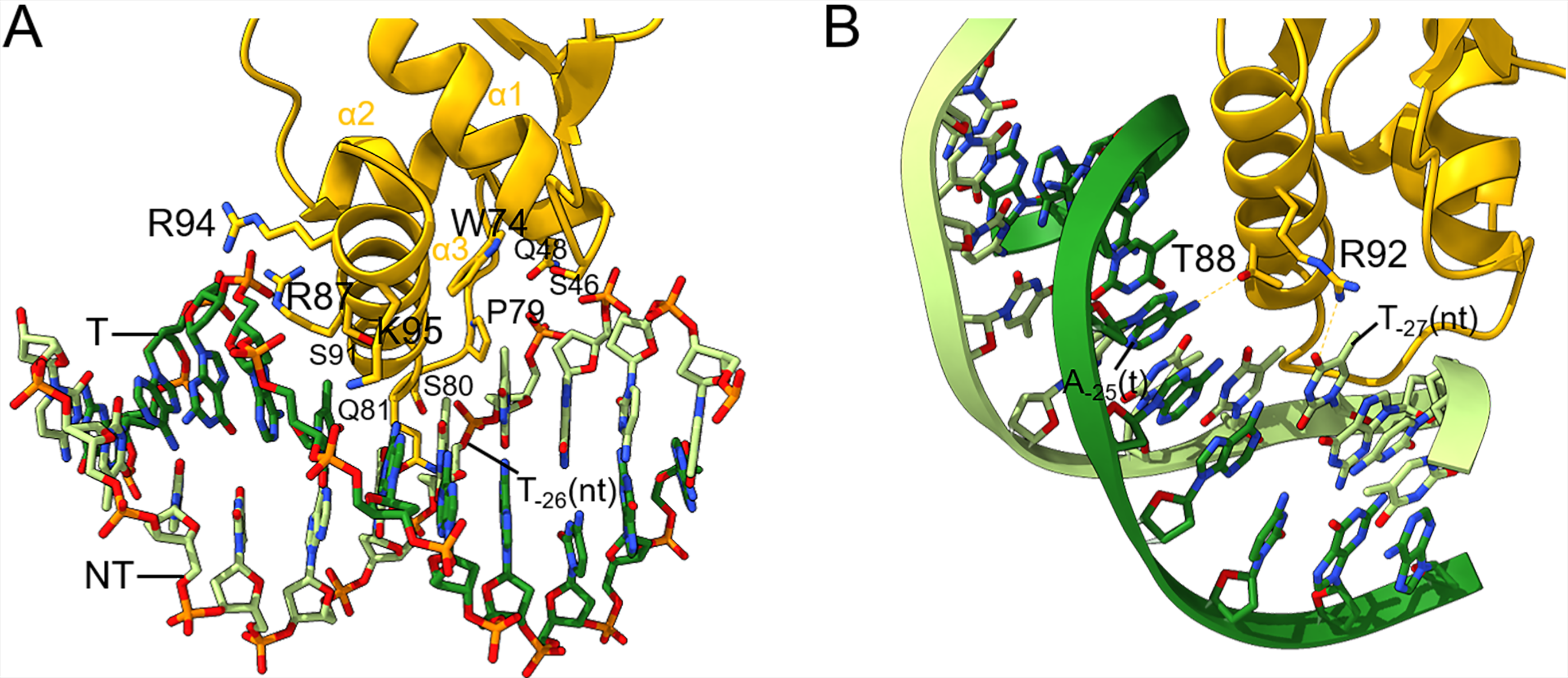

Supplement: S8 Fig — (A) Downstream SARP residues involved in interactions with the DNA backbone. (B) Contacts of downstream SARP with specific nucleotides. The residues R92 and T88 make hydrogen bonds (shown as yellow dashed lines) with T-27(nt) and A-25(t), respectively. (TIF) [file pbio.3002528.s008.tif]

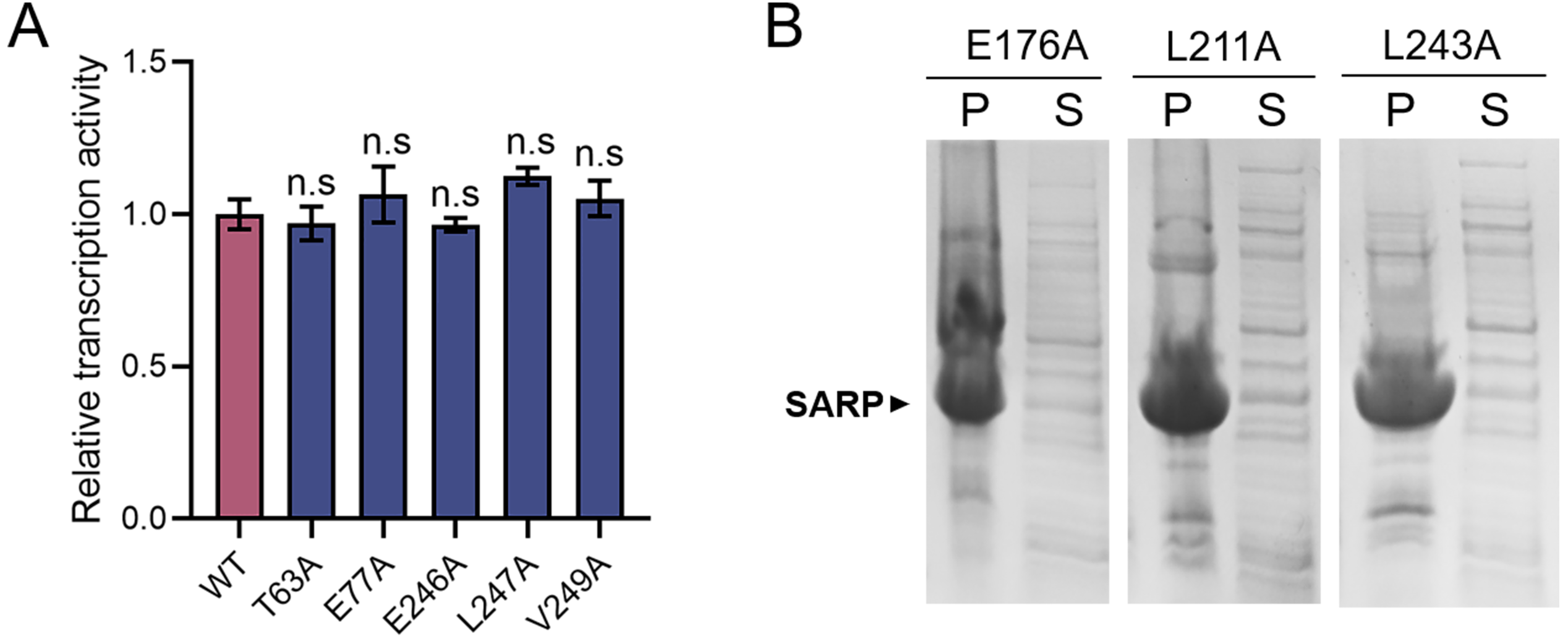

Supplement: S9 Fig — (A) In vitro transcription assays of SARP T63A, E77A, E246A, L247A, and V249A mutants. No obvious difference was observed between the T63A, E77A, E246A, L247A, V249A mutants, and the wild-type protein. n.s. means no significance. The data underlying this figure can be found in S1 Data; error bars, SEM; n = 3. (B) SDS-PAGE of E176A, L211A, and L243A mutants. Inclusion bodies were formed when these mutants were expressed under the same conditions as the wild-type protein. P represents precipitation while S represents supernatant. The original gel images can be found in S1 Raw Images. (TIF) [file pbio.3002528.s009.tif]

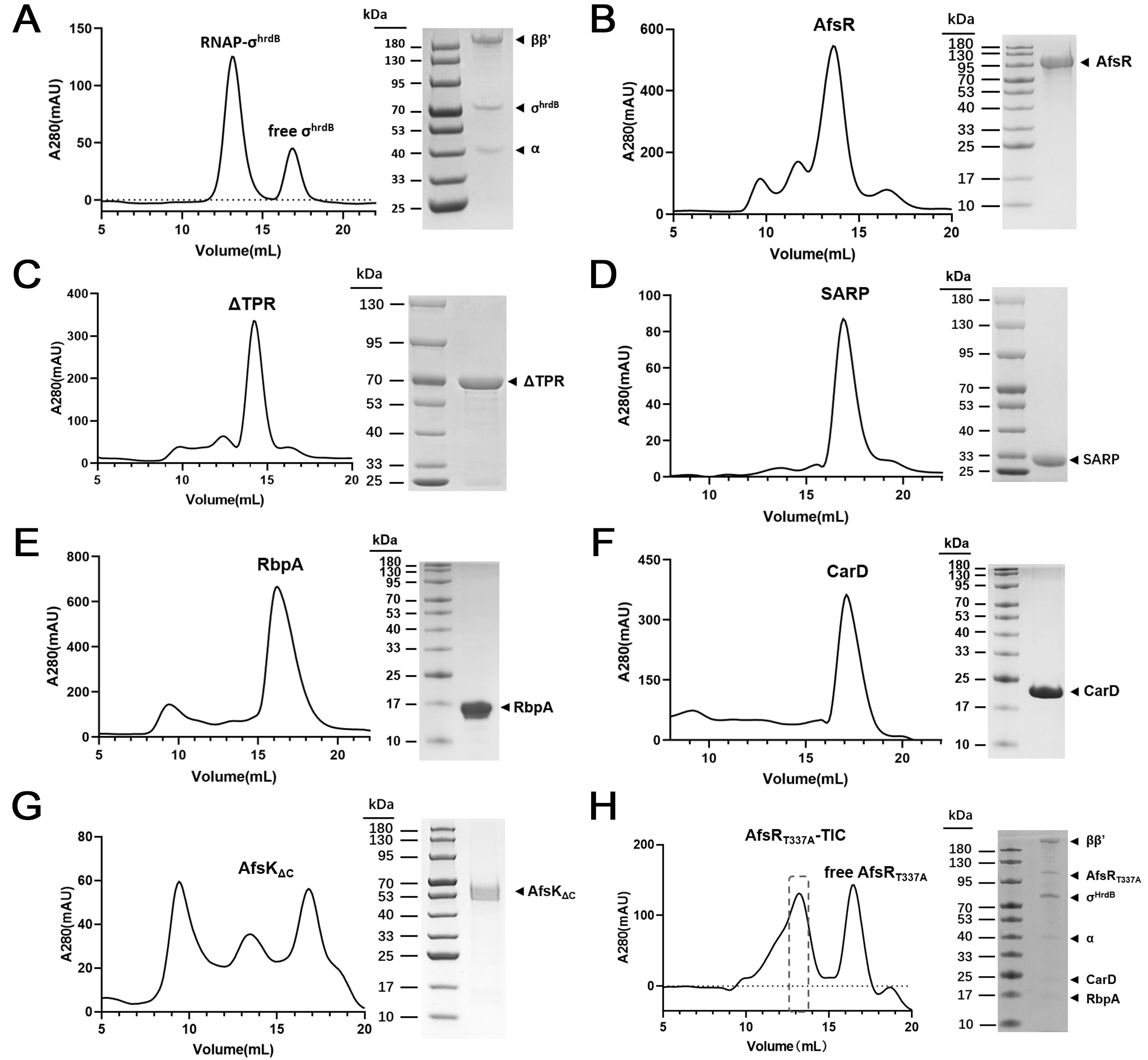

Supplement: S10 Fig — (A) Assembly of S. coelicolor RNAP-σHrdB. The peak of RNAP-σHrdB was analyzed by SDS-PAGE. (B) Purification of AfsR. (C) Purification of ΔTPR. (D) Purification of SARP. (E) Purification of RbpA. (F) Purification of CarD. (G) Purification of AfsKΔC. (H) Assembly of AfsRT337A-TIC in the presence of 1 mM ATPγS. The protein compositions in the dotted line boxed fractions are shown in the SDS-PAGE. The original gel images can be found in S1 Raw Images. (TIF) [file pbio.3002528.s010.tif]

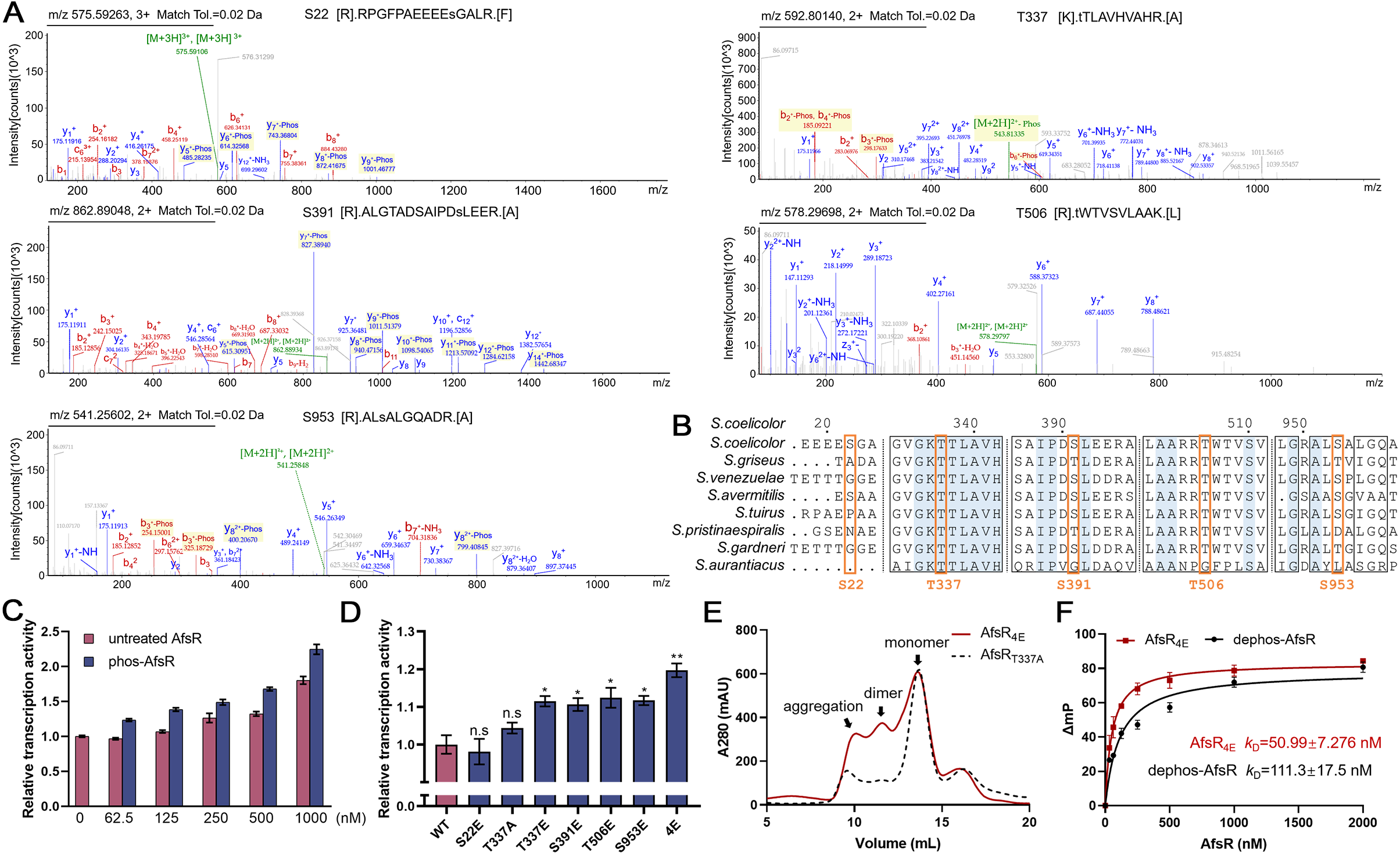

Supplement: S11 Fig — (A) LC-MS/MS analysis showing that S22, T337, S391, T506, and S953 are phosphorylated in AfsR by AfsKΔC. The lowercase letter in the peptide sequence indicates phosphorylated residue. “b” and “y” denote peptide fragment ions retaining charges at the N and C terminus, respectively. The subscript numbers indicate their positions in the identified peptide. (B) Sequence alignment of Streptomyces AfsR family members highlighting the consensuses sequences neighboring phosphorylation sites S22, T337, S391, T506, and S953. Orange boxes represent phosphorylation sites. (C) Transcription assays with increasing concentrations of phosphorylated AfsR by AfsKΔC (phos-AfsR) and untreated AfsR. (D) Transcription assays of 500 nM AfsR mutants mimicking dephosphorylation (T337A) and phosphorylation (S22E, T337E, T506E, S953E, and S391E/T337E/T506E/S953E (4E)). Data are presented as mean ± SEM from 3 independent assays. n.s. means no significance; *P < 0.05; **P < 0.01 in comparison with the wild-type AfsR. (E) Representative SEC assay of AfsRT337A and AfsR4E. (F) Fluorescence polarization assay of AfsR4E and dephosphorylated AfsR (dephos-AfsR) with afs box. The concentration of afs box was 10 nM. Error bars represent mean ± SEM of n = 3 experiments. The data underlying A, C, D, E, and F are provided in S1 Data. (TIF) [file pbio.3002528.s011.tif]

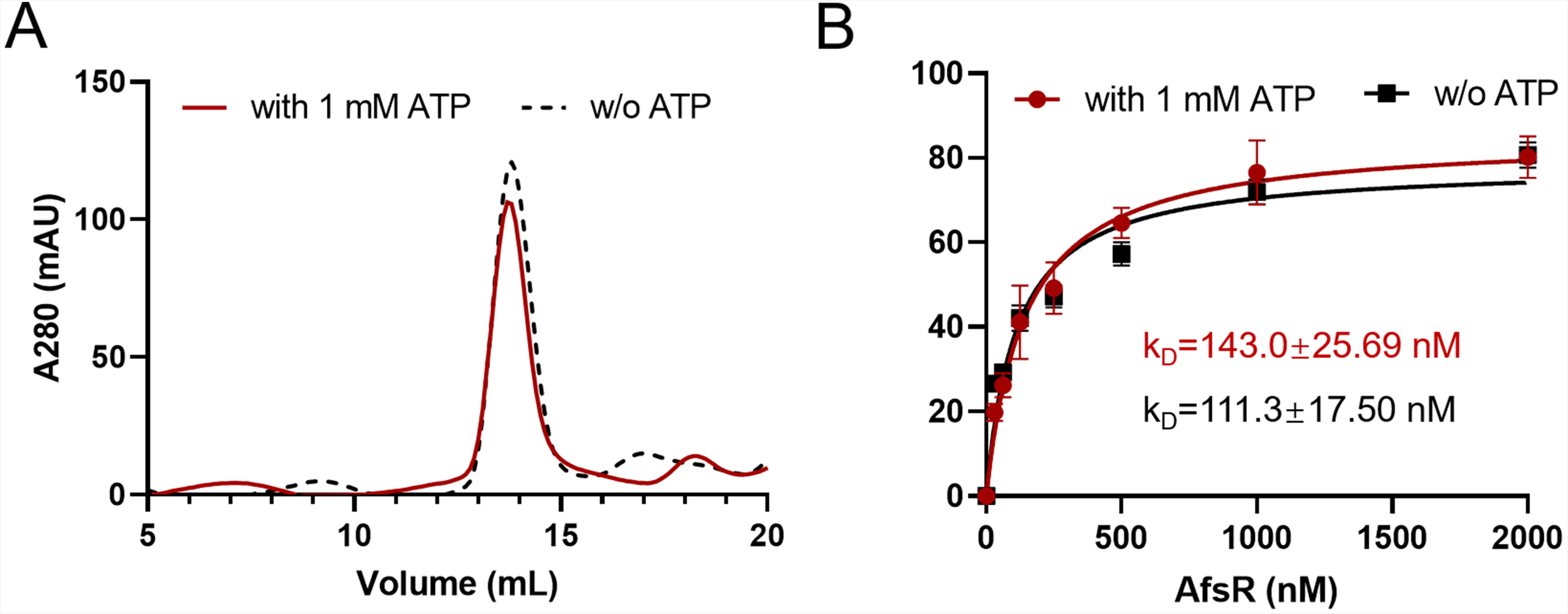

Supplement: S12 Fig — (A) SEC assay of dephosphorylated AfsR in the presence (solid line) or absence (dashed line) of 1 mM ATP. The data underlying this figure can be found in S1 Data. (B) Fluorescence polarization assay of dephosphorylated AfsR with afs box DNA in the presence or absence of 1 mM ATP. The concentration of afs box DNA was 10 nM. The data underlying this figure can be found in S1 Data; error bars, SEM; n = 3. (TIF) [file pbio.3002528.s012.tif]

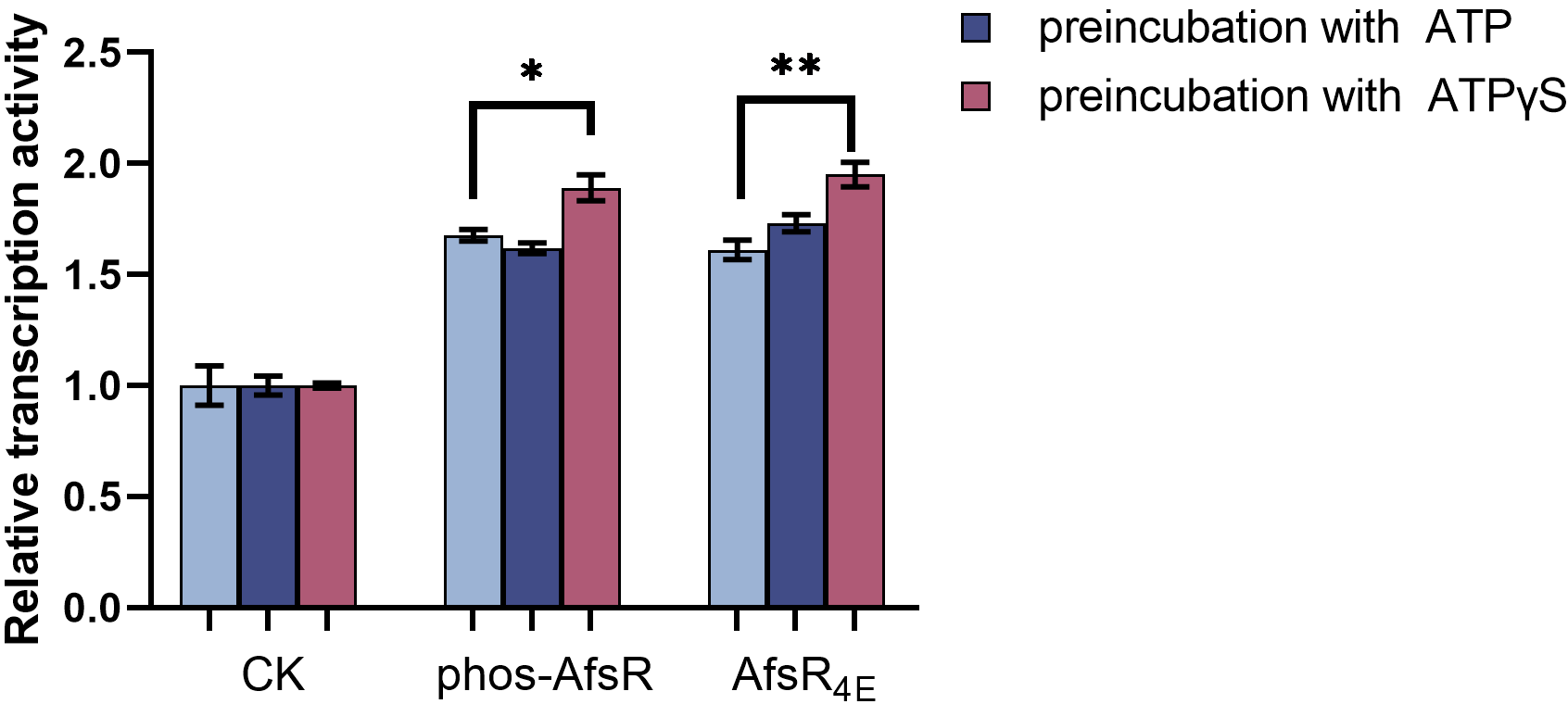

Supplement: S13 Fig — Transcription assays involving 500 nM phosphorylated AfsR (phos-AfsR) or AfsR4E, with and without preincubation with 1 mM ATP or ATPγS. CK represents the control group without the addition of AfsR. The data underlying this figure can be found in S1 Data; error bars, SEM; n = 3. (TIF) [file pbio.3002528.s013.tif]

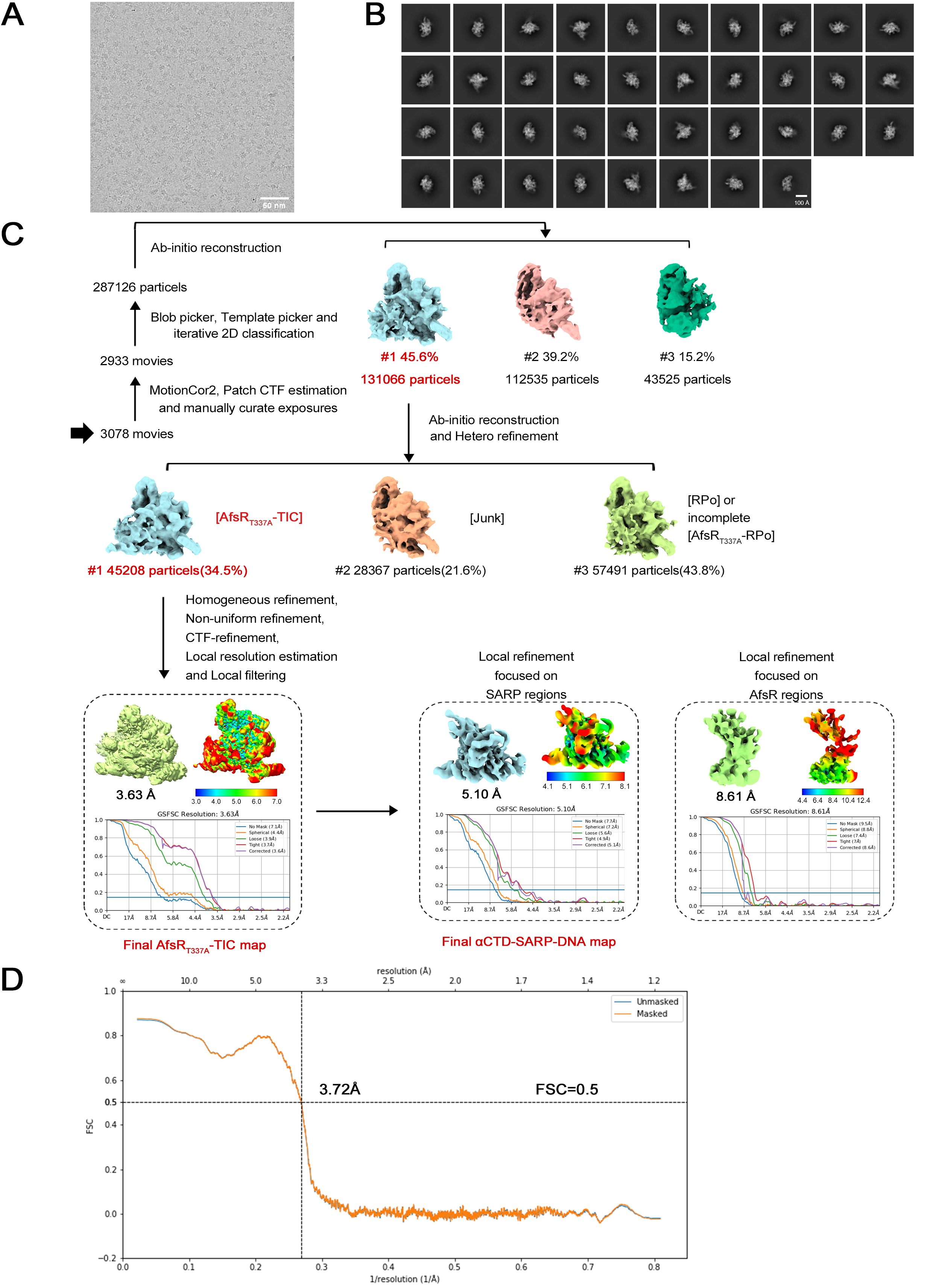

Supplement: S14 Fig — (A) Motion-corrected images (scale bar: 50 nm). (B) 2D classes (scale bar: 100 Å). (C) Data processing pipeline for the dataset of AfsRT337A-TAC. The final cryo-EM map of the AfsRT337A-TIC was reconstructed using a total of 45,208 single particles and refined to a nominal resolution of 3.63 Å. Local refinement focused on the SARP region generated a 5.10-Å-resolution map. Local refinement focused on the AfsR region generated a 8.61-Å-resolution map. (D) Validation of cryo-EM structural models. Map vs. model FSCs was generated by Phenix (Version 1.19.2). The model-map resolution for the atomic model and cryo-EM map at FSC = 0.5 cutoff was indicated in the figure and reported in S1 Table. The data underlying this figure can be found in S1 Data. (TIF) [file pbio.3002528.s014.tif]

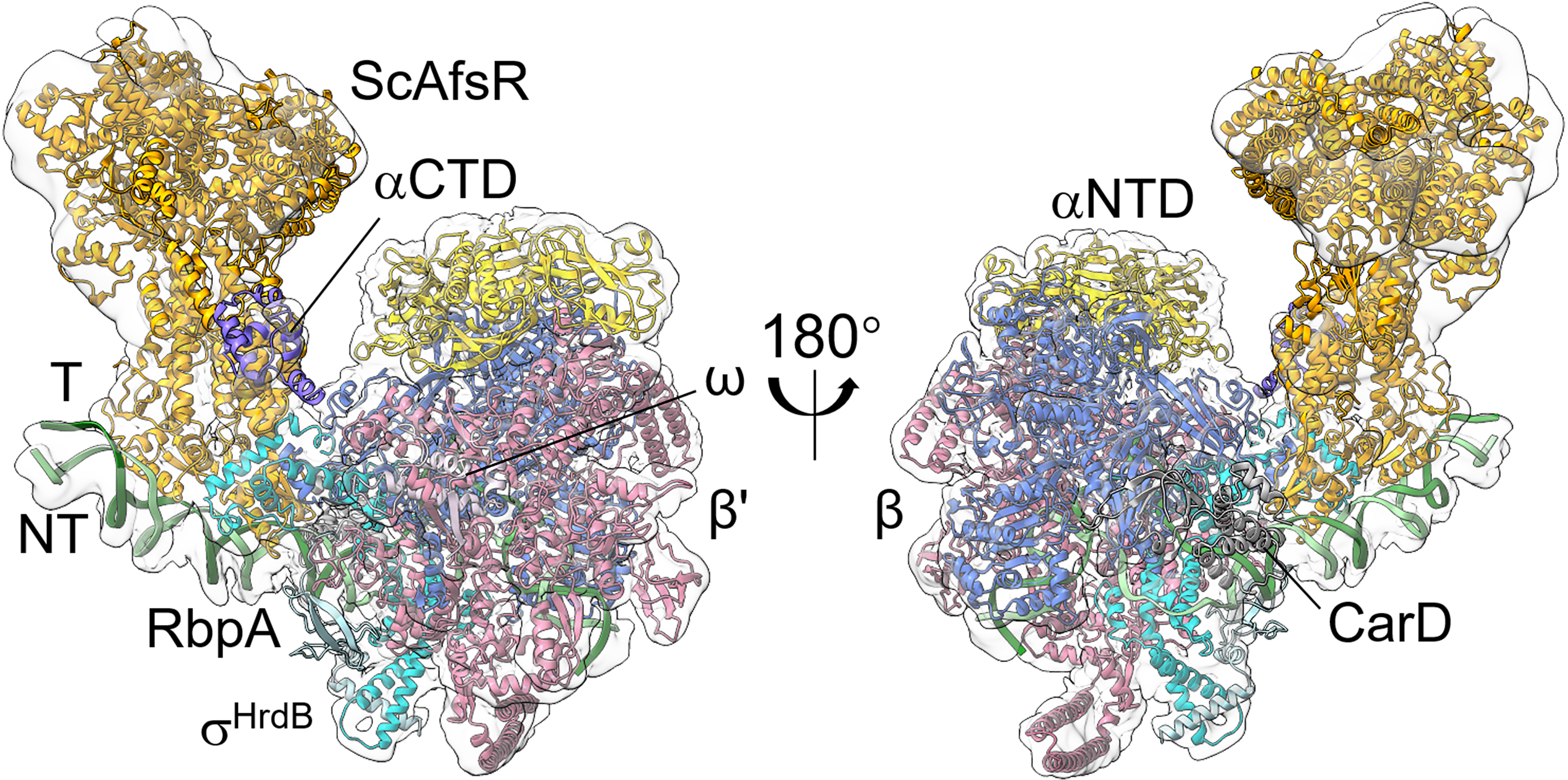

Supplement: S15 Fig — The map was generated by merging the consensus map of the full AfsR -TIC and the focused maps of the AfsR. The initial model of RNAP and AfsR was generated from SARP-TIC and AlphaFold, respectively, and fitted into the map using ChimeraX. (TIF) [file pbio.3002528.s015.tif]

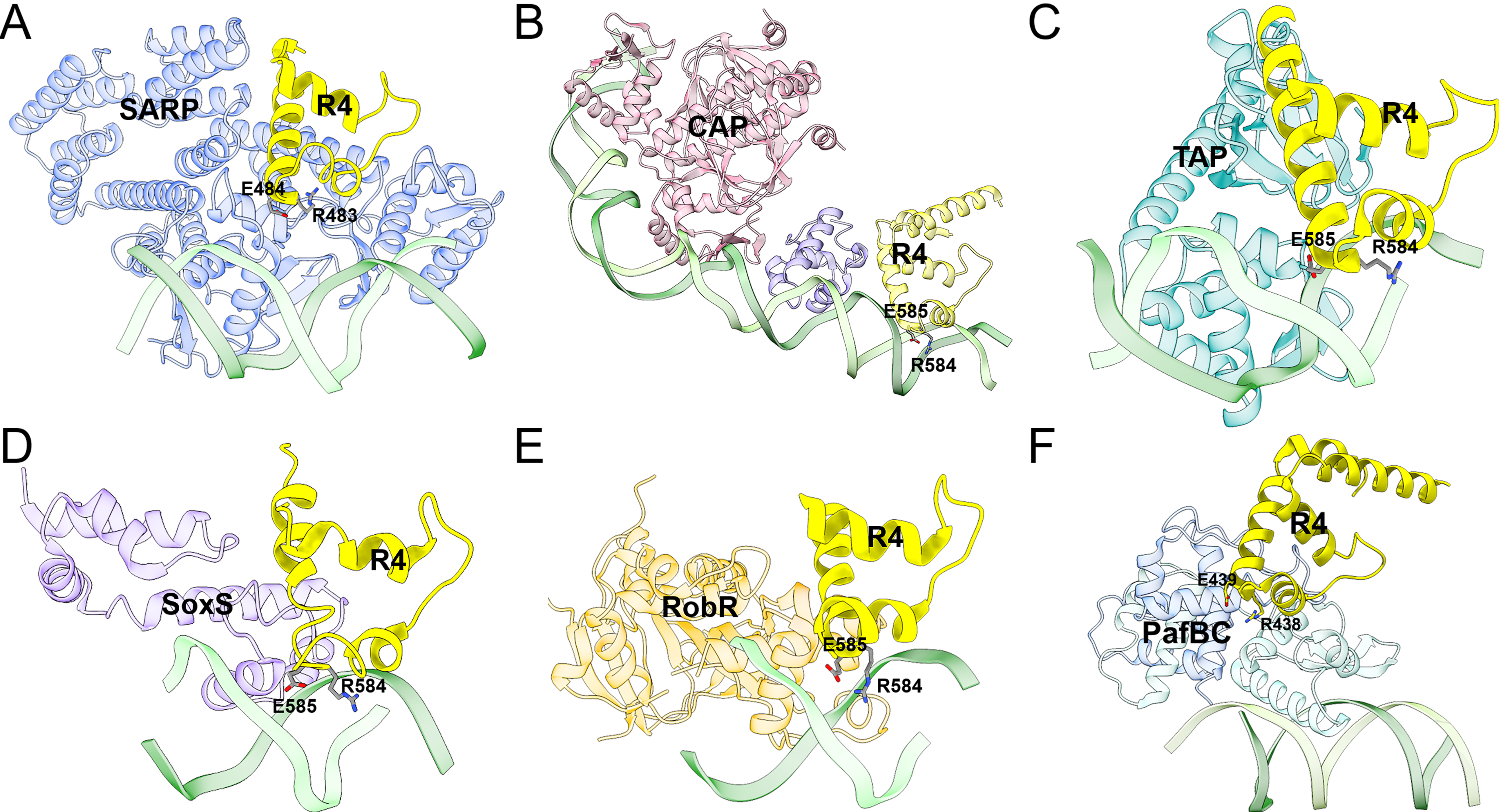

Supplement: S16 Fig — (A) SARP. (B) Class I CAP-transcription activation complex (TAC) (PDB ID:6B6H). (C) Class II TAP-TAC (PDB ID: 5I2D). (D) SoxS (PDB ID: 7W5W). (E) RobR (PDB ID: 7VWZ). (F) PafBC (PDB ID: 7P5X). The key residues Arg and Glu supposed to contact DNA are shown as sticks. (TIF) [file pbio.3002528.s016.tif]

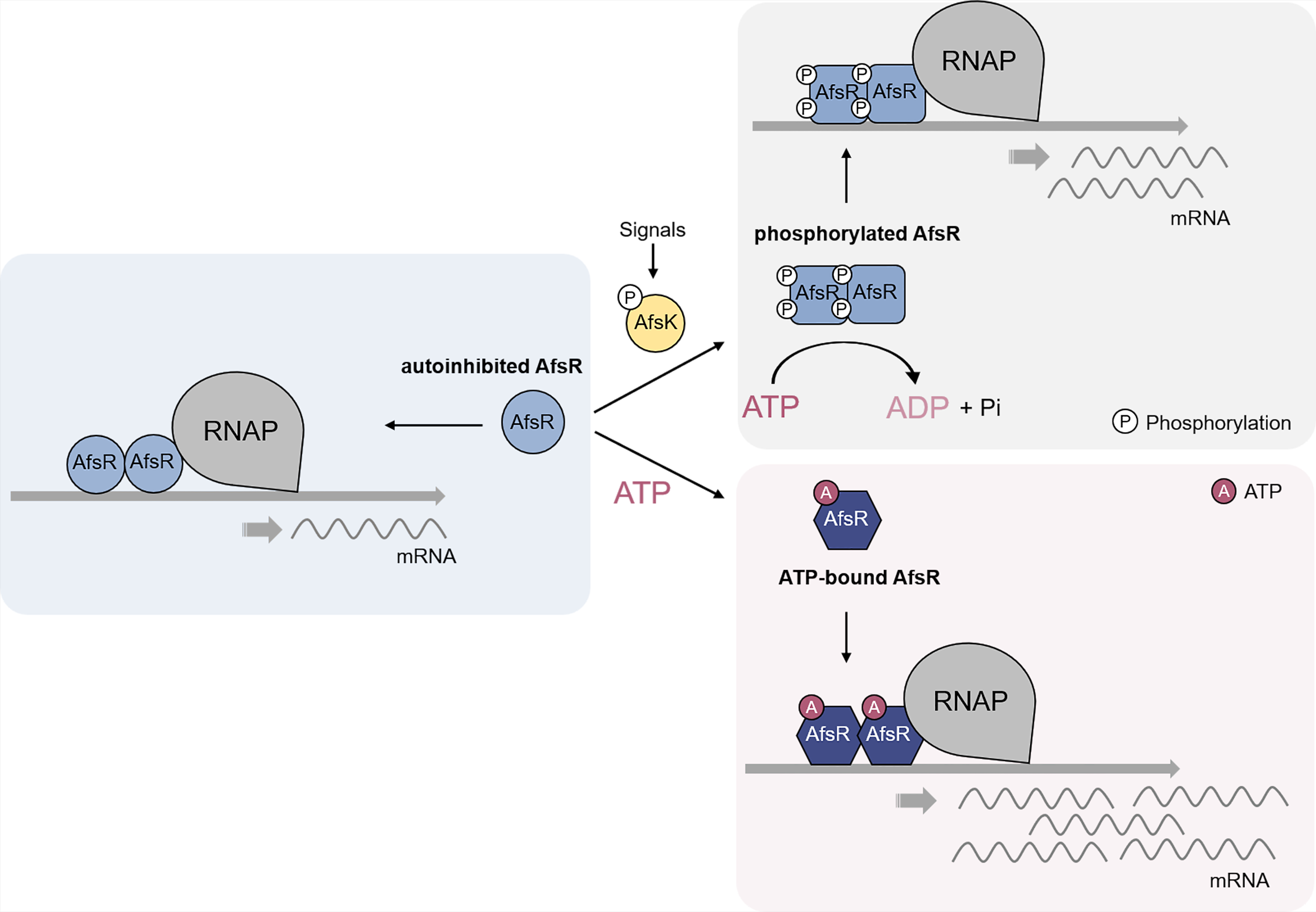

Supplement: S17 Fig — Unmodified full-length AfsR is autoinhibited by its C-terminal domains. AfsR is activated by ATP binding and significantly stimulates the production of transcripts. The phosphorylation of AfsR results in increased ATPase activity, potentially counteracting the effects of ATP binding. Phosphorylated AfsR forms more oligomers and shows slightly higher transcriptional activation compared to the unmodified form. (TIF) [file pbio.3002528.s017.tif]

**Fig. 1E**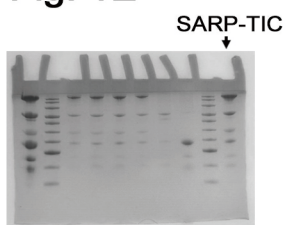**Fig. 5D****(a) dephos-AfsR**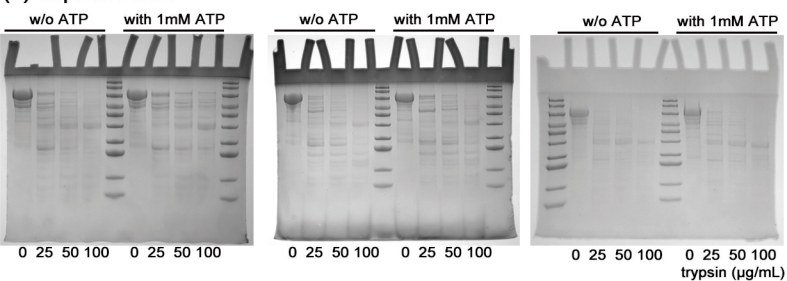**(b) AfsR<sub>T337A</sub>**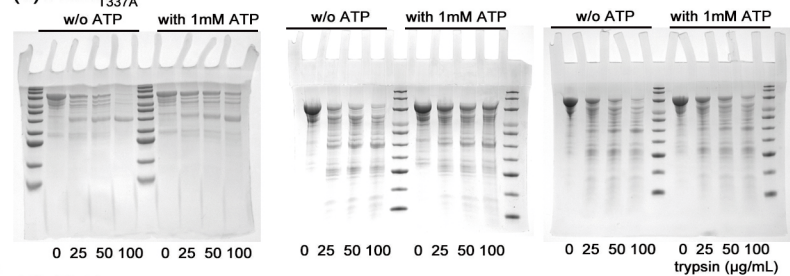**(c) phos-AfsR**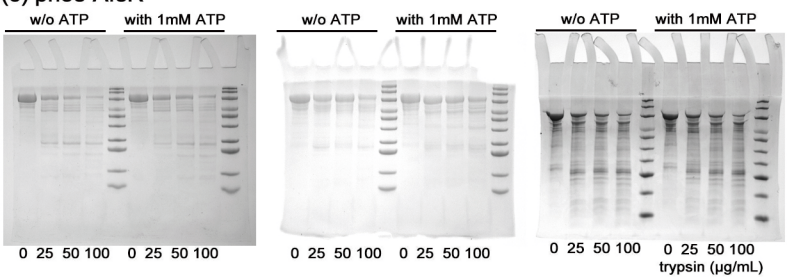**(d) AfsR<sub>4E</sub>**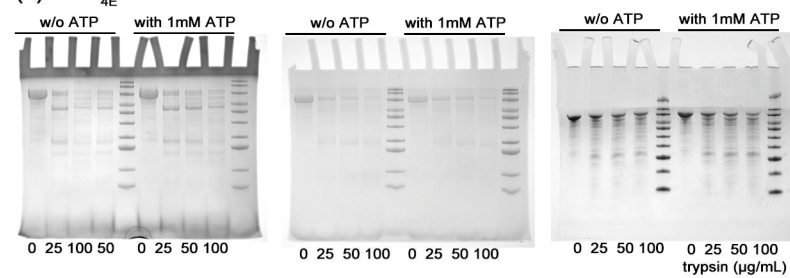**Fig. S9**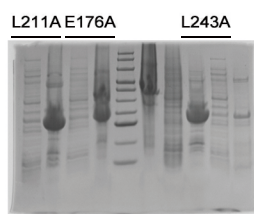**Fig. S10**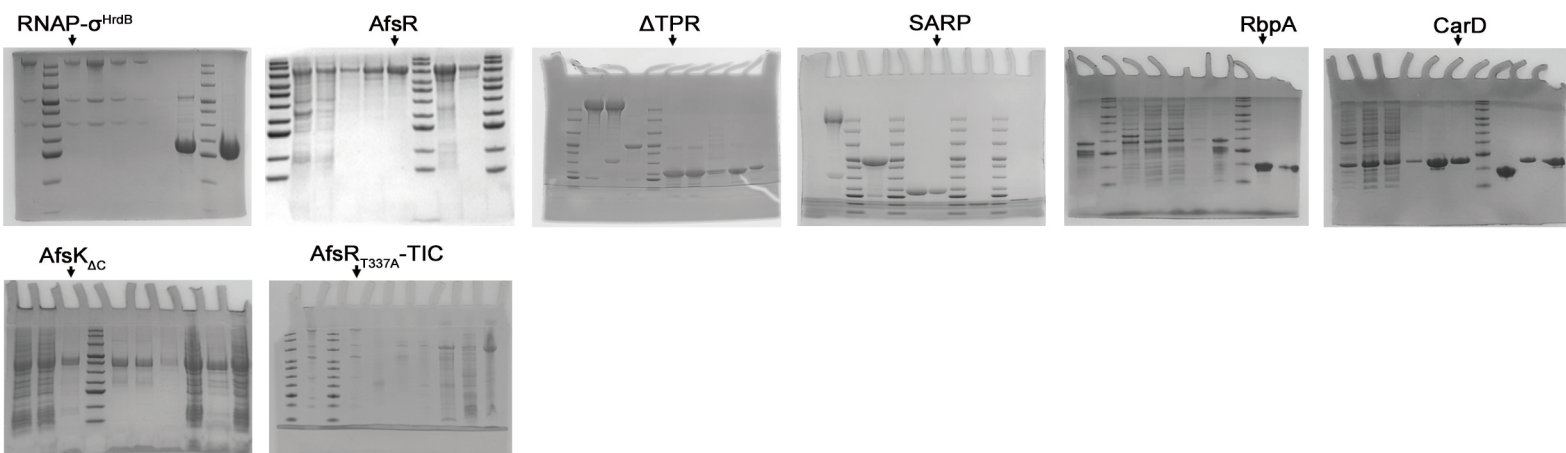

Supplement: S1 Raw Images — (PDF) [file pbio.3002528.s022.pdf]
