# Supplementary material for: Structural and functional characterization of AfsR, an SARP family transcriptional activator of antibiotic biosynthesis in Streptomyces
Source: PLoS Biol. 2024 Mar 1;22(3):e3002528. doi: 10.1371/journal.pbio.3002528 (PMC10936776; doi:10.1371/journal.pbio.3002528)
Supplement: Attachment — Submitted filename: Response to Reviewers.docx [file pbio.3002528.s023.docx]

Responses to Referees

Reviewer #1: In this paper, Wang and Zheng et. al. characterized the structure and mechanism of AfsR stimulated transcription. They determined high-resolution structure of Sarp domain of AfsR as well as full-length AfsR bound to transcription initiation complex (TIC), which together with biochemical assays illustrated how AfsR interacts with and stimulates transcription. In addition, biochemical investigation suggested a multi-layer regulation scheme of AfsR, which are duel controlled by ATP binding and phosphorylation. The paper presents significant new insights to Sarp-domain mediated transcription activation and could be general interests to broad audience. I only have several minor suggestions.

Response: Thank you very much for reviewing our manuscript. We sincerely appreciate the valuable suggestions. New supplementary figures have been prepared. The numbering of the supplementary figures has been updated accordingly. Please find the detailed responses below:

Minor:

1.        Scale bars are needed for the 2D classification images in Fig S2&10.

Response: Scale bars for the 2D classification images were added in Fig S3&14.

2.        Local densities of SARP domain and C-terminal domains of AfsR, possibly in FigS2&10 or in additional figures, are needed to show the quality of the maps, especially at the DNA binding region and C-terminal domains.

Response: Map and model fitting results at the DNA binding region were added in Fig 2A. Fig S15 illustrates the fitting of C-terminal domains of AfsR into the density. Fig S3C and Fig S14C display resolutions at the SARP and C-terminal domains of AfsR, respectively.

3.        A minor suggestion, possibly color the protein complexes and DNAs with different color in Fig S3 will be helpful to better illustrate the architecture of TICs.

Response: The protein complexes and DNAs were colored differently in Fig S4.

4.        In the TIC structure with SARP domain (Fig 2), the author claims the two protomer are nearly identical. A structural comparison will be helpful. In addition, protomer 1 has sequence specific DNA interactions and the promotor. How about protomer 2? Does it help with sequence recognition and transcription activation? In addition, do both protomers exert similar degree of DNA bending?

Response: A structural comparison of the two protomers has been included in Fig S5 to demonstrate their nearly identical structures. Two protomers make similar sequence specific interactions with DNA (Fig. S8, Page 7, Line 186-188). Biochemical assays presented in Figures S1B and S1C confirm the collaborative role of both protomers in sequence recognition and transcription activation. Mutational studies on the DRs, particularly at the downstream DR, significantly diminish AfsR's DNA binding and transcription activation capabilities (Page 5, Lines 111-119). Moreover, given the observed DNA bending at the midpoint between the two binding sites (T-30(nt)), it is presumed that both protomers equally contribute to the bending of the DNA (Page 7, Line 171-172).

5.  A scheme model to summarize the ATP dependent and phosphorylation dependent regulation of AfsR will be helpful.

Response: A scheme model illustrating the ATP-dependent and phosphorylation-dependent regulation of AfsR had been added in Fig S17.

Reviewer #2: This manuscript reveals some key aspects of how AfsR, a member of the Streptomyces antibiotic regulatory protein family binds at a target promoter to activate transcription, including some very interesting and novel contacts with various subunits of RNA polymerase. The work is of particular interest to anyone working with the regulation and expression of antibiotic biosynthetic clusters since AfsR is related to many key activators, and the structural model presented is likely to be broadly applicable to this family. The works is also of wider interest to the transcription field, showing another example of how flexibility in key RNAP subunits alpha-CTD and sigma can accommodate varied scaffolds that lead to activation. The work does not explore in any detail mechanistic questions of activation and the SARP role in the sequential process of initiation, but this is beyond the scope. Overall the presentation is very good and it reads very well. I have a few specific comments.

Response: Thank you very much for reviewing our manuscript and giving immensely valuable comments. We have carefully addressed each comment. Below are our detailed responses:

1.        The CACTGT -10 is close but not consensus since a T at the first position is most common. In fact it is argued in the paper that SARPs can compensate for poor -35 elements, however since most promoters have poor -35 elements, it is just as valid to suggest that at specific SARP-regulated promoters, the promoters lack activity in the absence of the SARP due to other "poor" promoter elements e.g. the -10 element.

Response: The diminished initiation activity of SARP-regulated promoters might result from multiple deficient promoter elements, such as the -10 region, the -35 region, and the spacer between the -10 and -35 regions. SARPs can potentially compensate for these deficient promoter elements. The corresponding discussion have been added in Page 3, Lines 78-79 and Page 12, Lines 333-334.

2.        Can the authors show that the in vitro transcription assays used are specific to transcription initiation at the afsS promoter rather than an increase in general activity of the RNAP as seen with end-to-end transcription on the DNA template. The assay with a control promoter would be useful here.

Response: Control experiments were conducted. SARP did not activate a control actⅡ-4 promoter that included a consensus −10 and −35 element but lacked the afs box (Fig. S1C, Page 5, Lines 114-117). Additionally, the ability of SARP to activate transcription was greatly diminished when the DRs were mutated (Fig. S1C, Page 5, Lines 117-119). These results showed specific transcription activation by SARP at the promoter having an intact afs box.

3.        Line 106. I think the authors mean disinhibition by ATP>

Response: Fixed (Page 4, Line 105).

4.        Line 114. The Fig 1 C/D are mislabelled.

Response: The mislabeling has been corrected.

5.        Line 115. It should be made clear that the SARP domain was used to build the TIC.

Response: We emphasized that the component used in TIC was the isolated SARP domain in Page 5, Line 120.

6.        Line 123. In the context of activation in Streptomyces it is important to know whether the SARP can activate transcription in the absence of RbpA and CarD. It seems an obvious omission and the paper would benefit from the inclusion.

Response: Fig. S1C showed that the wild-type SARP can significantly activate transcription in the absence of RbpA and CarD and was cited in Page 5, Line 119.

7.        The DNA scaffold used to form the TIC includes dsDNA around the -10 region, so it would be important to emphasise that the DNA is melted in the final TIC - ie that the complex has isomerized to the open complex.

Response: We emphasized that SARP-TIC is an open complex containing a transcription bubble of 13 nt in Page 5, Line 137.

8.        Line 292 and associated figure. The effect of ATP on limited proteolysis patterns of dephosphorylated AfsR vs phosphorylated AfsR is not that convincing and key differences could be highlighted. From my interpretation, the bigger difference appears to be between dephos-AfsR vs phos-AfsR rather than the effect of ATP.

Response: The key effect of ATP lies in enhancing the resistance of dephosphorylated AfsR and the AfsRT337A mutant against trypsin digestion (Page 11, Line 300), which is reflected in the observable reduction in the intensity of the primary AfsR bands. The major digestion bands of dephosphorylated AfsR and AfsRT337A were observed around 40 kDa, a feature no longer present in phosphorylated AfsR and the 4E mutant (Page 11, Line 306-309). The primary AfsR bands and major digestion bands were denoted by arrows and triangles in Figure 5D, respectively.

9.        Line 295 It is proposed that ATP hydrolysis might explain why the phosphorylated AfsR is not activated by ATP binding - what happens when a non-hydrolysable analogue of ATP is used?

Response: The transcription assays with a non-hydrolysable ATP analogue were conducted, and the result showed that phosphorylated AfsR, along with the 4E mutant, exhibits partial activation in response to non-hydrolysable ATPγS (Page 11, Lines 304-305, Fig. S13).

10.        Line 331 The phrase that SARP functions as an adaptor to physically bond the -35 and sigma R4 is a bit ambiguous and might suggest that sigma makes normal contacts with -35 that are held in place by SARP. In fact the SARP provides an alternative docking point for R4 meaning that a -35 region is not required.

Response: The description in Page 12, Line 343 has been refined to convey that SARP provides an alternative docking point for σ R4.
